# No recovery in the biomass of flying insects over the last decade in German nature protected areas

**DOI:** 10.1002/ece3.11182

**Published:** 2024-03-24

**Authors:** Roland Mühlethaler, Sebastian Köthe, Thomas Hörren, Martin Sorg, Lisa Eichler, Gerlind U. C. Lehmann

**Affiliations:** ^1^ NABU (The Nature and Biodiversity Conservation Union) Berlin Germany; ^2^ Entomological Society Krefeld (EVK) Krefeld Germany; ^3^ Leibniz Institute of Ecological Urban and Regional Development (IOER) Dresden Germany; ^4^ Evolutionary Ecology Humboldt University Berlin Berlin Germany

**Keywords:** agriculture, biodiversity loss, insect biomass, insect decline, malaise traps, natura 2000, nature conservation

## Abstract

Five years after a German study on insect biomass described a multi‐decade decline in nature protected habitats, the DINA (Diversity of Insects in Nature protected Areas) project has investigated the status of insects in 21 selected nature reserves across Germany in the years 2020 and 2021. We used the same methods and protocols for trapping and measuring the biomass of flying insects as in the earlier study. Across two vegetation periods, we accumulated a comprehensive data set of 1621 data points of two‐week emptying intervals to evaluate the insect biomass along gradients from arable land into nature reserves through transects of Malaise traps. On average, we observed an increase in maximum insect biomass per day along the transect from the edge to the centre of the nature reserve. Overall, the measured insect biomass remained at low levels, consistent with previous findings from the years 2007–2016. There were no significant regional differences. The results show that protected habitats have higher insect biomass compared to farmland and are therefore essential for insects but are unlikely to be sufficient to sustain insect biodiversity. Further measures need to be taken for better protection and sustainment of insects, which fulfil key functions in all terrestrial ecosystems.

## Graphical abstract

Across two seasons, we accumulated a comprehensive data set on insect biomass from representative nature reserves across Germany. The measured overall insect biomass remained at low levels and corresponds to the published latest figures from 2007 to 2016. There were no significant regional differences, but biomass was negatively correlated with agricultural production area within 2 km of nature reserves.

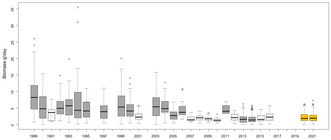

## 1 INTRODUCTION

In the past few decades, alarming declines of insects have been observed in many places all over the world (Burner et al., 2021; Powney et al., 2019; Seibold et al., 2019). The study of Hallmann et al. (2017) illustrated the decline of insect biomass in nature protected areas in Germany over 27 years. The discussions this study generated made a significant contribution to raising public and political awareness of the problem of insect decline. Since then, several studies provided evidence that insect biomass decline involves a loss in diversity (Hallmann, Ssymank, Sorg, de Kroon, & Jongejans, 2021; Hallmann, Ssymank, Sorg, Kroon, & Jongejans, 2021; Hausmann et al., 2022). In addition, habitat fragmentation is a major driver of insect decline and its negative impact on insect populations is a major problem in many regions (Köthe, Bakanov, Brühl, Eichler, et al., 2023; Köthe, Bakanov, Brühl, Gemeinholzer, et al., 2023; Köthe, Schneider, Bakanov, Brühl, et al., 2023; Tscharntke et al., 2002; Tscharntke & Brandl, 2004). Due to their species richness, their biomass and their diverse specialisations, insects play a key role in almost all ecosystems. Insects are essential for the pollination of many wild plants and crops, or the transformation and decomposition of organic material (soil formation and fertility). They are also important food sources for other animals such as birds, fish and bats. Therefore, the loss of insects significantly degrades ecological networks and leads to a decline in ecosystem services. This has potentially serious consequences for society and the economy (Baur et al., 2020; Conrad et al., 2004).

Nature protected areas (NPAs: category IV according to IUCN Protected Areas Categories System; Lausche & Burhenne‐Guilmin, 2011) serve as legally binding areas for the special protection of nature and landscape in their entirety or in parts in order to enable the conservation, development or restoration of habitats, biotopes or communities of certain wild animal and plant species. Therefore, the protection of local biodiversity should have unrestricted priority for all biotope types and land uses, including arable land. The German red list of threatened habitat types (Finck et al., 2017) shows that without exception all arable habitat types, including a high degree of the wild arable plant communities, are classified as ‘threatened with complete extinction’. A similarly dramatic situation is evident for wildlife in these arable habitats. With agricultural use, as a major cause of insect decline (Sánchez‐Bayo & Wyckhuys, 2019), NPAs and especially special areas of conservation (SAC) are vitally important for the preservation of wild flora and fauna species and their natural habitats at the European level (Natura 2000 network). However, further research is needed to measure the influence of agricultural practices on the observed insect decline and to develop and implement biodiversity‐enhancing practices (Eichler et al., 2022; Sorg et al., 2019).

DINA is a comprehensive interdisciplinary research project involving eight institutions (Lehmann et al., 2021), investigating insect communities and the influence of arable farming on them in 21 NPA in Germany. All selected NPAs are directly adjacent to farmland and part of SACs within the European Natura 2000 network (Brühl et al., 2021; Eichler et al., 2022; Köthe, Bakanov, Brühl, Eichler, et al., 2023; Lehmann et al., 2021; Swenson et al., 2022). Based on the original model to study the main drivers for the decline in insect biomass (Hallmann et al., 2017), we focused on already identified covariates: the influence of arable land and the chemical inputs such as nitrogen and pesticides associated with intensive land use. In contrast to Hallmann et al. (2017), which was an analysis covering only a geographically limited region in Germany, our study was comprehensively conducted at comparable sites (NPA) across Germany covering most biogeographical regions and investigating different scientific questions, such as landscape elements and their spatial distribution pattern (Eichler et al., 2022), the diversity of plant communities (Köthe, Bakanov, Brühl, Gemeinholzer, et al., 2023; Swenson et al., 2022), pesticide residues (Brühl et al., 2021) and insect biomass along gradients from arable land into protected habitats through transects (Brühl et al., 2021; Köthe, Bakanov, Brühl, Eichler, et al., 2023). In addition, this natural science approach is accompanied by co‐development with local stakeholders of solutions for improved insect protection (Fickel et al., 2020; Köthe, Bakanov, Brühl, Eichler, et al., 2023; Lehmann et al., 2021).

We present the biomass data of two consecutive years 2020 and 2021 from 1621 Malaise trap samples in 21 NPAs across Germany. As we applied almost identical methods as those used the Entomological Society Krefeld (Hallmann et al., 2017; Hallmann, Ssymank, Sorg, de Kroon, & Jongejans, 2021; Hallmann, Ssymank, Sorg, Kroon, & Jongejans, 2021; Sorg et al., 2019; Ssymank et al., 2018), a direct comparison with previous data is possible to allow a continuation and update for insect biomasses within NPA across Germany.

## 2 MATERIALS AND METHODS

### 2.1 Sampling sites and Malaise trap setup

**FIGURE 1:**
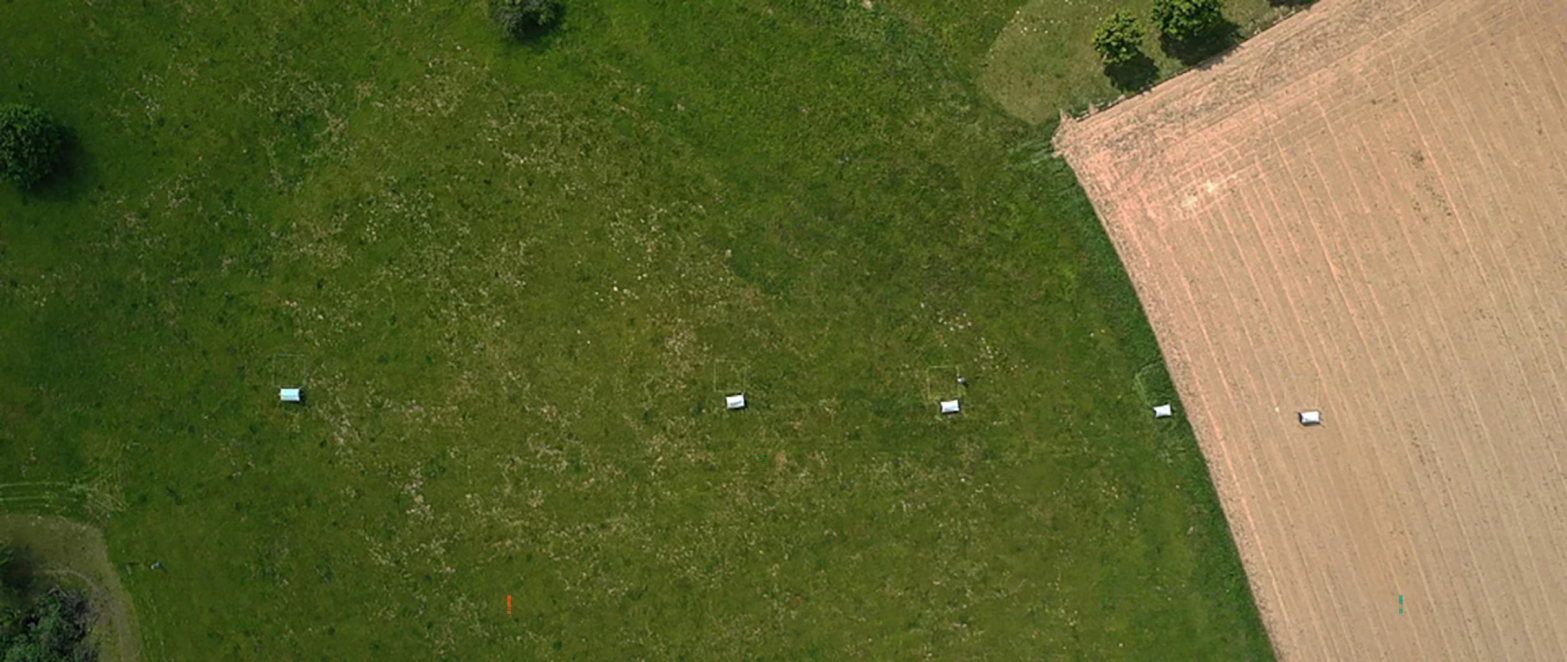
Aerial view of a Malaise trap transect starting with MT1 on arable land (right in picture), MT2 at the boarder of the protected habitat and on the left MT5 towards the centre of the nature reserve (copyright: Entomological Society Krefeld).

Based on spatial analyses, landscape indicators were first evaluated, resulting in a pre‐selection of sampling sites from a total of 8836 nature reserves in Germany (Eichler et al., 2022; Lehmann et al., 2021). Approximately 11,033 km of farmland is directly adjacent to NPAs. In the case of SACs, this contact line is approximately 21,102 km (Eichler et al., 2022). Only sites that fulfilled our requirements for high‐quality grassland‐dominated habitat types of Natura 2000 (Table S1) with adjacent or integrated arable land and representing most German natural areas remained in the pre‐selection. To identify arable land, an GIS analysis was performed using the Land Cover Model Germany LBM‐DE 2018 (BKG 2018; Meinel & Reiter, 2019). In addition, the cooperation of local authorities and landowners was another criterion for the final selection of the sites. Malaise traps have proven to be a reliable method for effective sampling of insects (Sorg et al., 2019). In 2020 and 2021, Malaise traps were set up in 21 nature reserves located within the borders of special areas of conservation (SAC) as part of the European Natura 2000 network across Germany to investigate insect communities from early May to late August with collection intervals of 2 weeks (Table S2). Five Malaise traps were set at each site, creating a transect that started on arable land (Malaise trap 1 = MT1), continued along the boundary between agricultural land and protected area (MT2) and extended into the protected area (MT3–MT5), with approximately 25 m between each trap (Figure 1; see also Lehmann et al., 2021). At some sites, landowners withdrew their consent to place a Malaise trap on their fields, even during the current sampling period. At four sites in both years and at a further three in 2021, the first trap was therefore relocated at the boundary between agricultural land and protected area, corresponding to Malaise trap 2 (MT2a and MT2b in those cases). At one site (Brauselay, Rhineland‐Palatinate), the first trap had to be completely removed in 2021 because the local situation did not allow relocation to alternative spots.

Comparability of data with past and future insect monitoring studies was ensured by using the standardised Malaise traps (type Townes, 1972) of the Entomological Society Krefeld (Sorg et al., 2019; Ssymank et al., 2018). These traps collect flying insects in 1000‐mL polyethylene bottles, preserved in 96% ethanol from which biomass was determined (Hallmann et al., 2017; Ssymank et al., 2018). The ethanol containing the catches of insects and other arthropods is poured over a stainless‐steel fine sieve (mesh size <0.5 mm), which allowed unwanted by‐catches such as snails (Pulmonata) to be sorted out. After the frequency of the ethanol drop exceeds approx. 10 s, the fresh biomass is measured on a fine balance (T500Y, G&G GmbH Neuss, lower measuring accuracy <0.1 g). Animals and ethyl alcohol are then returned to the original polyethylene bottles. Because trapping intervals varied in some cases by a few days we standardised the wet biomasses by dividing the total wet biomass of each interval by the number of days the traps were active, resulting in a mean of calculated (but not measured) daily biomass (Hallmann et al., 2017).

### 2.2 Climate data

Data on maximum temperature and daily precipitation were obtained from the German Weather Service (DWD). For each site, the closest weather station (distances ranging from 2.5 to 22.8 km from the Malaise trap sites) was identified to collect information. As the indicator for the overall climate conditions, we used the continentality index provided by the DWD Climate Data Centre (2021) for each study site. The continentality indicates the strength of the influence of land masses on the climate. In Germany, continentality increases from northwest to southeast with greater temperature fluctuations between summer and winter as well as day and night as you move to the southeast. The biogeographic and natural regions are strongly related to the continentality.

### 2.3 Statistical analysis

Statistical analysis was carried out using Rx64 4.0.1 (R Core Team, 2019).

Our methodology is unique in applying transects of traps, including borders and adjacent arable land, in the sampling scheme. To compare our data with previous similar studies conducted in German NPA, especially the landmark data by Hallmann et al. (2017), we restricted several analyses to our traps located in protected habitats. For these analyses, the data of the traps MT3–MT5 located within the NPA were pooled into a single mean. Since the data for the continentality index and the agricultural production area within 2 km did not differ for MT3–MT5, the daily biomass of traps was averaged for traps MT3–MT5. The average maximum temperature and average precipitation were calculated accordingly. Generalised additive models (GAMs) were used to analyse the effects of maximum temperature (ranging from 20.2–24.4°C in 2020 to 19.7–23.9°C in 2021), precipitation (ranging from 1.0–3.3 mm/day in 2020 to 1.4–4.2 mm/day in 2021), continentality index (ranging from 15.5 to 19.0) and agricultural production area within 2 km (ranging from 0.5 to 9.2 km^2^, with an average of 4.7 km^2^) on daily biomass using the ‘mgcv’ package fitted with the Restricted Maximum Likelihood (REML) method (Wood, 2011, 2017). The code for the model was structured as follows (example for the year 2020): mod = gam (dailybiomass_2020 ~ s (Temperature_2020, *k* = 3) + s (Precipitation_2020, *k* = 3) + s (Continentality_index, *k* = 3) + s (Arable_land_2km, *k* = 3), data = x, method = “REML”, family = gaussian). For the complete R code, see Table S3.

Due to significant changes in land use (e.g. newly established wild flower strips, flowering fallows) adjacent to four NPA sites (Riedensee, Insel Koos, Bottendorfer Hügel and Schwellenburg), we performed two different GAM analyses per year. Four factors (temperature, precipitation, continentality index and agricultural production area within 2 km radius) were tested as predictive variables of insect biomass in four generalised additive models (GAMs), separated between years 2020 and 2021 and either including MT3–MT5 within NPA from all 21 localities or excluding the four sites with landscape change between years (Table S4).

For an analysis of local insect biomass dominance, we categorised the transect according to which trap location had the highest observed mean biomass based on the median values (see Figure S1) into four classes: highest mass on (i) *arable land* (AL at MT1), (ii) *border* (Bor at MT2: traps directly at the boundary of the nature protected area), (iii) within the *nature protected areas* (NPA with MT3–MT5) and (iv) *indistinguishable* (Ind, at least two trap classes show similarly high values).

## 3 RESULTS

**FIGURE 2:**
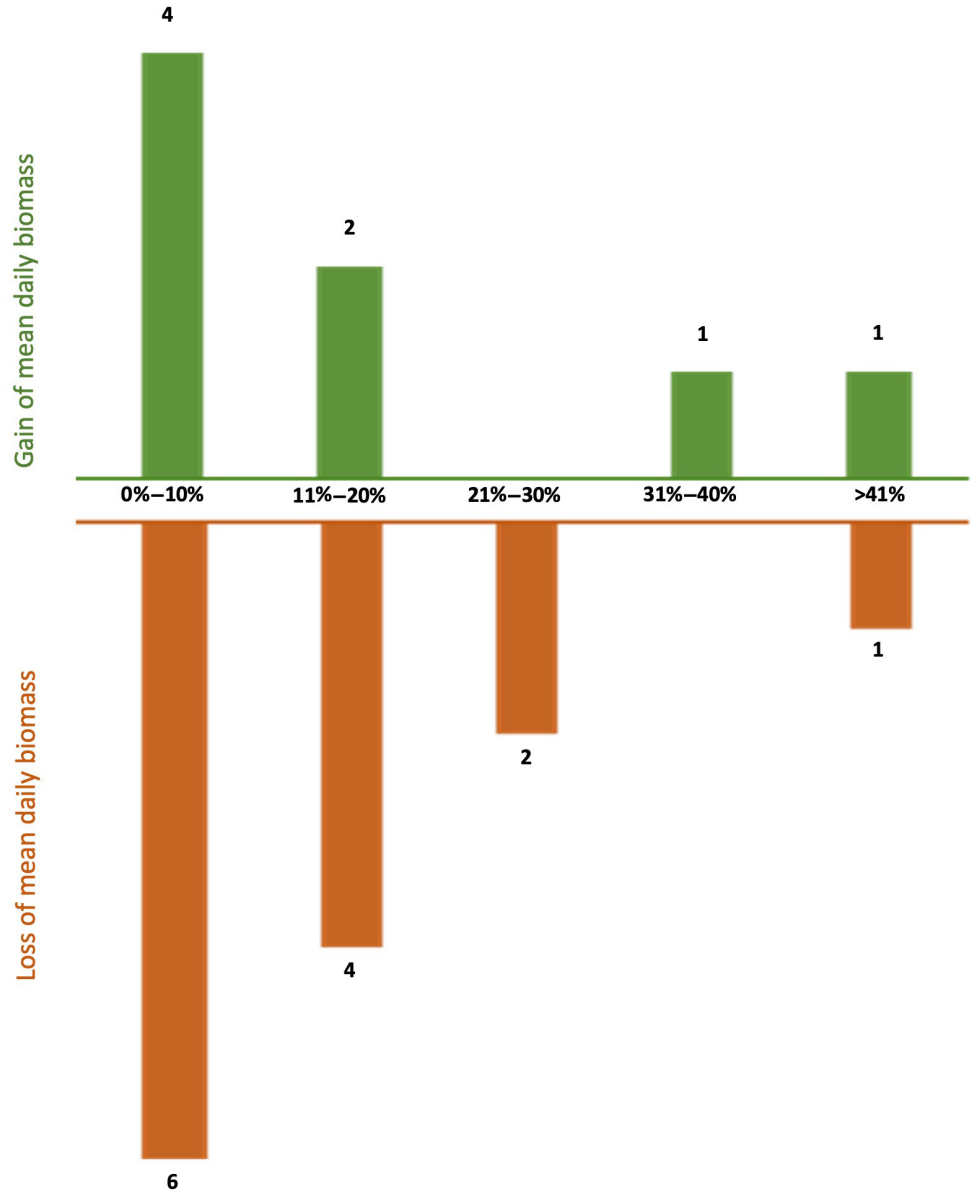
Number of locations with increase (green) or decrease (orange) of mean daily biomass between the two monitoring periods 2020 and 2021 (the percentage indicates the difference in biomass).

Late spring and summer insect biomass varied from 0.95 (SE ± 0.47) to 3.62 (SE ± 1.98) grams per day, based on 1621 samples for both years (829 from 2020 and 792 from 2021; Table S5). From year to year, these biomasses fluctuate considerably at the same locations (Figure S1). At 13 sampling sites, we found a decrease in insect biomass and at eight sites an increase from 2020 to 2021 (Table 1; Figure 2).

**TABLE 1:** Areas categorised by the trap position with the highest insect biomass within their transect for the years 2020 and 2021: Highest mean biomass on *arable land* (AL), *border* between arable land and nature protected area (Bor), within *nature protected area* (NPA), or *indistinguishable* (at least two trap classes show similarly high values) (Ind).

Sites	2020	2021	Difference in biomass 2020/2021 (in %)
Lütjenholmer Heidedünen	Bor	Bor	−8.8
Riedensee	NPA	AL	+51.4
Insel Koos	NPA	AL	−2.7
Geesower Hügel	Bor	Bor	+11.2
Oderhänge Mallnow	AL	AL	+20.0
Wisseler Dünen	NPA	Bor	−10.6
Bislicher Insel	NPA	Ind	+4.7
Gipskarstlandschaft Hainholz	Ind	Bor	−19.3
Porphyrlandschaft bei Gimritz	Ind	Bor	−19.9
Ziegenbuschhänge bei Oberau	NPA	NPA	−4.5
Wipperdurchbruch	Ind	Ind	−1.0
Bottendorfer Hügel	Bor	Ind	−7.1
Schwellenburg	Bor	Bor	+30.1
Hofberg	NPA	NPA	−22.8
Koppelstein‐Helmestal	NPA	Ind	+4.4
Rheinhänge Dörscheider Heide	Bor	Ind	+5.1
Brauselay	NPA	NPA	−21.2
Mittelberg	Ind	Ind	−42.4
Ipf	Bor	Bor	−17.9
Kürnberg	Ind	Ind	+6.1
Mühlhauser Halde	Ind	Ind	−6.7

*Note*: Categorised dominance patterns found unchanged between the year 2020 and 2021 are indicated in blue.

When categorising areas by the trap position with the highest mean biomass within their transect (at *arable land* [AL], the *border* between arable land and nature protected area [Bor], within *nature protected areas* [NPA], or *indistinguishable* with similarly high values for at least two categories [Ind]), the annual repeatability was moderately high with 12 out of 21 sites showing similar dominance patterns for both years 2020 and 2021 (Table 1; Table S6).

The shift in 2021 of six traps from arable land to the borders due to farmers withdrawal of consent had little impact on the biomass dominance distribution, as in five cases the pattern was identical to the previous year 2020 and only in one case shifted the highest biomass from an indistinguishable distribution (Ind) towards the border (Bor). Therefore, the bias due to relocation of traps from arable land in the second year seems to be small. The highest biomass occurred equally in the NPA or at the border, or showed no clear pattern, classified as indistinguishable (Table 1; Table S6).

Despite this general pattern, we observed quite a number of deviations due to the local situation. In one case, the insect biomass was higher on arable land, at a site with environmentally friendly farming and consequently a species‐rich vegetation, including many endangered plants (for details see Köthe, Bakanov, Brühl, Gemeinholzer, et al., 2023; Table 1: Oderhänge Mallnow). Changes in land use have also been found to alter biomass from 1 year to the next: in two cases, the highest mean biomass shifted in the second year (2021) from NPA to previous arable land, which can be attributed to newly established wildflower strips. Furthermore, flowered fallow land and uncut ‘flowering islands’ around Malaise traps, led to a shift in biomass distribution from 1 year to the next, particularly pronounced at two sites (Table 1; Figure S1).

**FIGURE 3:**
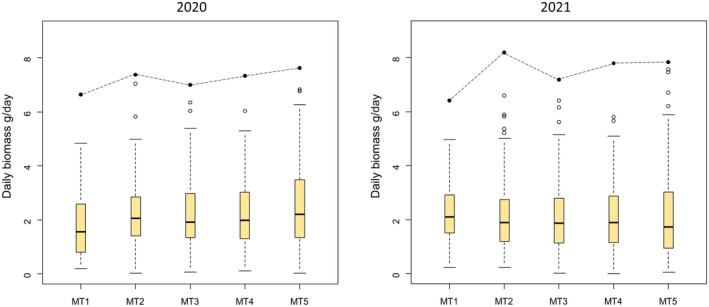
Boxplots (median, interquartile range, whiskers and outliers) of daily insect biomass along the Malaise trap transect (MT1–MT5). Sampling year 2020 on the left, and 2021 on the right. Full circles connected by a dashed line at the top represent the maximum biomass.

Overall, the insect biomass per day in 2020 was highest at the most central point within a NPA along the transect (MT5) with a median of 2.21 g/day, and the highest maximum biomass per day with 7.62 g/day (Figure 3 left). The situation was different in 2021. Due to newly established wildflower strips, flowered fallow land and uncut ‘flowering islands’ around four of the Malaise traps (MT1), the original experimental setup was distorted. The insect biomass was highest on arable land (MT1) with a median of 2.05 g/day and the maximum biomass of 8.19 g/day was highest at the border (MT2; Figure 3 right).

**FIGURE 4:**
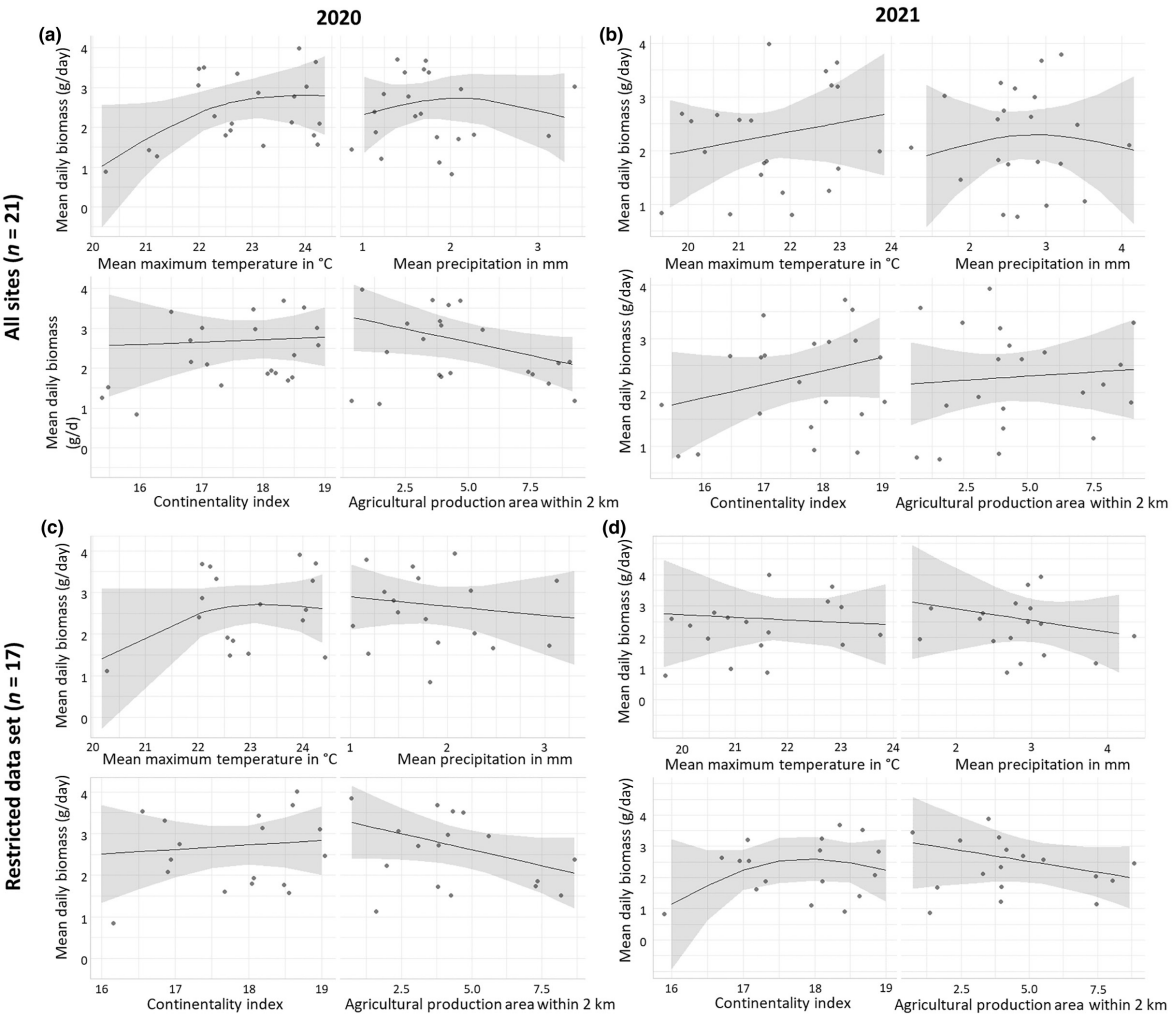
Results of generalised additive models (GAMs) that examine the effects of mean maximum temperature, mean precipitation, continentality index and agricultural production area (2 km radius around NPA) on mean insect biomass per day within NPA (pooled data from MT3 to MT5). Model a includes all 21 sites for 2020, and model b for 2021. Model c excludes four sites with newly established ‘flower strips’ (Riedensee, Insel Koos, Bottendorfer Hügel, Schwellenburg) for 2020, and model d for 2021.

To enable a better comparison with previous similar studies in German NPA, we have limited the following analyses to the traps MT3–MT5 (with a total of 500 samples from 2020 and 475 from 2021). These traps were located within the protected areas, while traps on borders and in adjacent arable land were excluded. Generalised additive models revealed that three indices of climate (mean maximum temperature, mean precipitation and continentality index) were not associated with insect biomass (Figure 4a–d; Table S7). Agricultural production areas within 2 km of NPAs tended to be negatively associated with insect biomass in 2020 (*p* = .07; Figure 4a) but not in 2021 (*p* = .72; Figure 4b). Excluding the four sites with newly established ‘flower strips’ in 2021, the agricultural production area within a radius of 2 km had no significant negative effect on the mean daily insect biomass in both years (2020: *p* = .10, Figure 4c; 2021: *p* = .32, Figure 4d).

**FIGURE 5:**
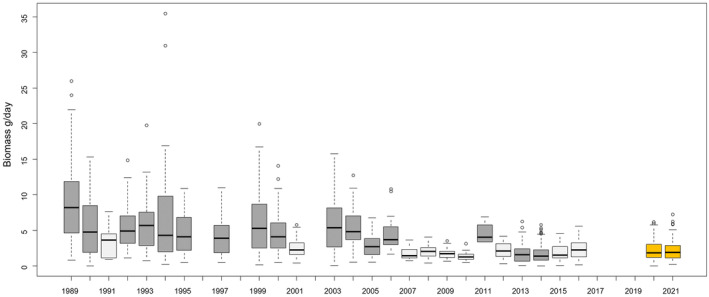
Daily insect biomasses in gram in German nature protected areas (NPA) of a 27‐year timeseries published in Hallmann et al. (2017) (dark grey = significantly different to 2020 and 2021; light grey = no significant difference to 2020 and 2021, Mann–Whitney *U* test) compared to our data (mean daily biomass of MT3–MT5) from 2020 to 2021 (orange). Some years had small numbers of samples (1991 *n* = 10, 2011 *n* = 4 and 2015 *n* = 10).

The Malaise traps were emptied 189 times across the 21 sites in 2020 and 165 times in 2021. The samples cover various biogeographical regions in Germany, including potentially more species‐rich areas in southern Germany, according to the comprehensive data from Entomofauna Germanica (Klausnitzer, 2003). Despite selecting high‐quality NPAs with endangered plant communities and biotope types according to the Red Lists for Germany, our measurements show a comparable low level of biomass according to the data from the earlier period (2007–2016; Hallmann et al., 2017; Figure 5, for statistical details see Table S8).

## 4 DISCUSSION

In general, our study shows a relatively low biomass of flying insect in 21 NPA spread across Germany. Insect biomass levels were similarly low as during the last years in the previous study by Hallmann et al. (2017) over a period of 27 years. While using the same standardised methods and traps, Hallmann et al. (2017) focused on NPA mainly in three German federal states, two in the west (North Rhine‐Westphalia, Rhineland Palatinate) and one in the east (Brandenburg). In contrast, we selected NPAs, which represent the different biogeographical and natural regions across Germany (Lehmann et al., 2021), including some in warmer climates in southern Germany, where insect species richness is generally higher (Klausnitzer, 2003). We did not find significant regional differences among the 21 sites. In line with another recent study using a different type of Malaise traps (Welti et al., 2022), we also observed that mean maximum temperatures correspond positively but not significantly with trapped biomass. The differences between the two monitoring periods of 2020 and 2021 were considerable in some sites (Table 1; Figure 2). Fluctuations of insect populations are a well‐known natural phenomenon and one of the major challenges in interpreting insect monitoring data (Didham et al., 2020).

A recent publication by Müller et al. (2023) claimed that measured insect biomass has increased significantly in recent years and has already returned to the level observed 30–40 years ago. However, a critical analysis of their methods finds Müller et al.'s conclusion to be potentially biased, due to selective sampling, statistical inference that is not consistent with conventional model selection, and the validation data rely more on within year weather variation (Hallmann et al., submitted rebuttal letter). In addition, similar but not identical methods have been used, and it has been shown by Uhler et al. (2022) that different types of Malaise traps produce different biomass results. Furthermore, it was shown that landscape‐level drivers and insect decline are associated with each other (Seibold et al., 2019).

The absence of a recovery in insect biomass reported in this paper is consistent with another German study (Hörren et al., 2023) that also used the same methods as Hallmann et al. (2017).

On average, we observed an increase in maximum insect biomass per day along the transect from the edge towards the centre of nature protected areas in 2020 and from MT3 to MT4 in 2021. With only 2 years of data, no general trend of increase or decrease in insect biomass can be derived by our results. However, due to the methodology, the data are directly comparable with the data from Hallmann et al. (2017) and show a similar low level of insect biomass for entire Germany. The reasons for the deviations in biomass observed by us between the two consecutive years seem to be diverse and might be influenced by variation in land‐use practices and other anthropogenic impacts. After the first year of the DINA project, some farmers changed their habits and started planting flower strips near our traps to support insects, changed crop management, or stopped agricultural cultivation (see Table S5). We could observe immediate consequences at two sites, where flower strips were established in 2021, possibly in response to the project. In both cases, the maximum insect biomass was shifted from protected habitats towards the previously cultivated arable land (Table 1; Figure S1) and can be explained by the change of local land use around this trap. A flowered fallow was created at the Bottendorfer Hügel site in 2021, while the vegetation around MT 1 and 2 was not cut at the Schwellenburg site in 2021, so that distinctive flowering islands have formed around the Malaise traps (Figure S2). In both cases, we could register shifting effects on insect biomass distribution along the transect. In the first year, the maximum biomass was close to the centre of the NPA, while in the second year, the maximum was at the edge or even outside the NPA. Consistent with our results, other studies have shown that flower strips have a major impact on insect abundance, increasing the number of individuals and species (Jönsson et al., 2015; Lowe et al., 2021). However, the insects move from their natural habitats into the nearby arable land, potentially increasing the risks of exposure to insecticides and other pesticides (Botías et al., 2016; Brühl et al., 2021; Hahn et al., 2015). These measures and effects were considered in our calculations. The model, which excluded the sites with such measures, showed an association between the proportion of arable land within a radius of 2 km around NPA. Different field management methods can also influence the occurrence of insects. Maize, for example tends to result in higher arthropod biomass compared to other cultivations (Frizzas et al., 2018; Hüber et al., 2022; Musters et al., 2021; Sorribas et al., 2016). The site with the most stable conditions between 2020 and 2021 with almost no changes in insect biomass (−1%; Table 1, Table S5, Figure S1) was recorded at the Wipperdurchbruch, probably due to the fact that the NPA has no direct contact with arable land. These findings emphasise our previous results on fertiliser and pesticide drift into the NPA, which documented strong chemical edge effects and negative impacts from adjacent arable land on plant communities in nature protected areas caused by intense agricultural practices. In addition, the number of endangered plant species rose with increasing proximity to the edge of the field (Köthe, Bakanov, Brühl, Gemeinholzer, et al., 2023).

Although higher biomass prevailed in the NPA, one‐third of our sites had the maximum measured biomass at the borders. This can be related to edge effects, as many flying insects patrol along prominent terrain boundaries. Such boundaries can be the break between relatively tall cereals and low natural vegetation, as is often seen at field edges. But even differences that are not visible to humans, such as differences in plant species compositions, can be perceived by insects as borderlines (Macfadyen & Muller, 2013; Nguyen & Nansen, 2018). Insects attracted by these boundaries stay there for longer periods and if the fields are chemically treated, they face a higher risk to be contaminated with insecticides and other pesticides. Few sampling sites had the maximum insect biomass on arable land. At the Oderhänge Mallnow, environmentally friendly farming practices over the years have led to a high diversity of plants, including endangered species that are typical for arable land (Köthe, Bakanov, Brühl, Gemeinholzer, et al., 2023).

## 5 CONCLUSIONS

Our study confirms the low level of insect biomass in German nature protected areas compared to data from two to three decades ago. Furthermore, our results show that nature reserves act as important habitats for insects, with maximum biomasses closer to the centre of these areas. Nevertheless, they are often not sufficiently buffered against negative influences from directly adjacent, conventionally farmed croplands. Appropriate measures must therefore be taken to better implement the conservation objectives of nature reserves and their habitats, as these areas are often the last refuges of critically endangered species.

Changes in land use such as newly established flower strips and bee friendly cultivations as measures to improve the situation for insects seemed to have an immediate impact in our study. Increased edge effects are seen at the immediate borders of cultivated areas. Many insects patrol along these, and this behavioural pattern of highly mobile species brings large numbers of insects close to pollution sources, which contradicts nature conservation goals. Therefore, conservation measures must be planned and linked to other accompanying actions to provide the insects with the best possible food and reproduction opportunities and protect them from exposure to pesticides.

The decline in insect biomass in German nature reserves shows that insects, which perform key functions in our ecosystems, need better protection and that efforts so far have not been able to reverse their loss.

## AUTHOR CONTRIBUTIONS

**Roland Mühlethaler:** Conceptualization (equal); investigation (equal); project administration (equal); writing – original draft (lead); writing – review and editing (lead). **Sebastian Köthe:** Data curation (equal); formal analysis (equal); investigation (equal); project administration (equal); validation (equal); visualization (equal); writing – original draft (equal); writing – review and editing (equal). **Thomas Hörren:** Investigation (equal); methodology (equal); writing – review and editing (equal). **Martin Sorg:** Conceptualization (equal); funding acquisition (equal); investigation (equal); methodology (equal); writing – review and editing (equal). **Lisa Eichler:** Investigation (equal); visualization (equal); writing – review and editing (equal). **Gerlind U. C. Lehmann:** Conceptualization (equal); funding acquisition (lead); investigation (equal); methodology (equal); project administration (lead); writing – original draft (equal); writing – review and editing (equal).

## FUNDING INFORMATION

The Project DINA is funded by the German Federal Ministry of Education and Research (BMBF) and is handled by the VDI Project Management Agency (grant number FKZ 01LC1901). Conceptual framework and development of methodologies of the Entomological Society Krefeld (EVK) were funded by the German Federal Ministry for the Environment, Nature Conservation, Nuclear Safety and Consumer Protection (BMUV), handled by the Bundesamt für Naturschutz (BfN), grant number FKZ 3516850400.

## CONFLICT OF INTEREST STATEMENT

The authors declare that they have no conflict of interest.

## Supporting information


Appendix S1.

## Data Availability

Our data will be freely accessible via the IOER Research Data Centre: https://ioer‐fdz.de/en/.
