# Supplementary material for: No recovery in the biomass of flying insects over the last decade in German nature protected areas
Source: Ecol Evol. 2024 Mar 24;14(3):e11182. doi: 10.1002/ece3.11182 (PMC10961242; doi:10.1002/ece3.11182)
Supplement: Supplementary file 1 — Appendix S1. [file ECE3-14-e11182-s001.docx]

***Manuscript “No recovery in the biomass of flying insects over the last decade in German nature protected areas”***

**Supplement**

**
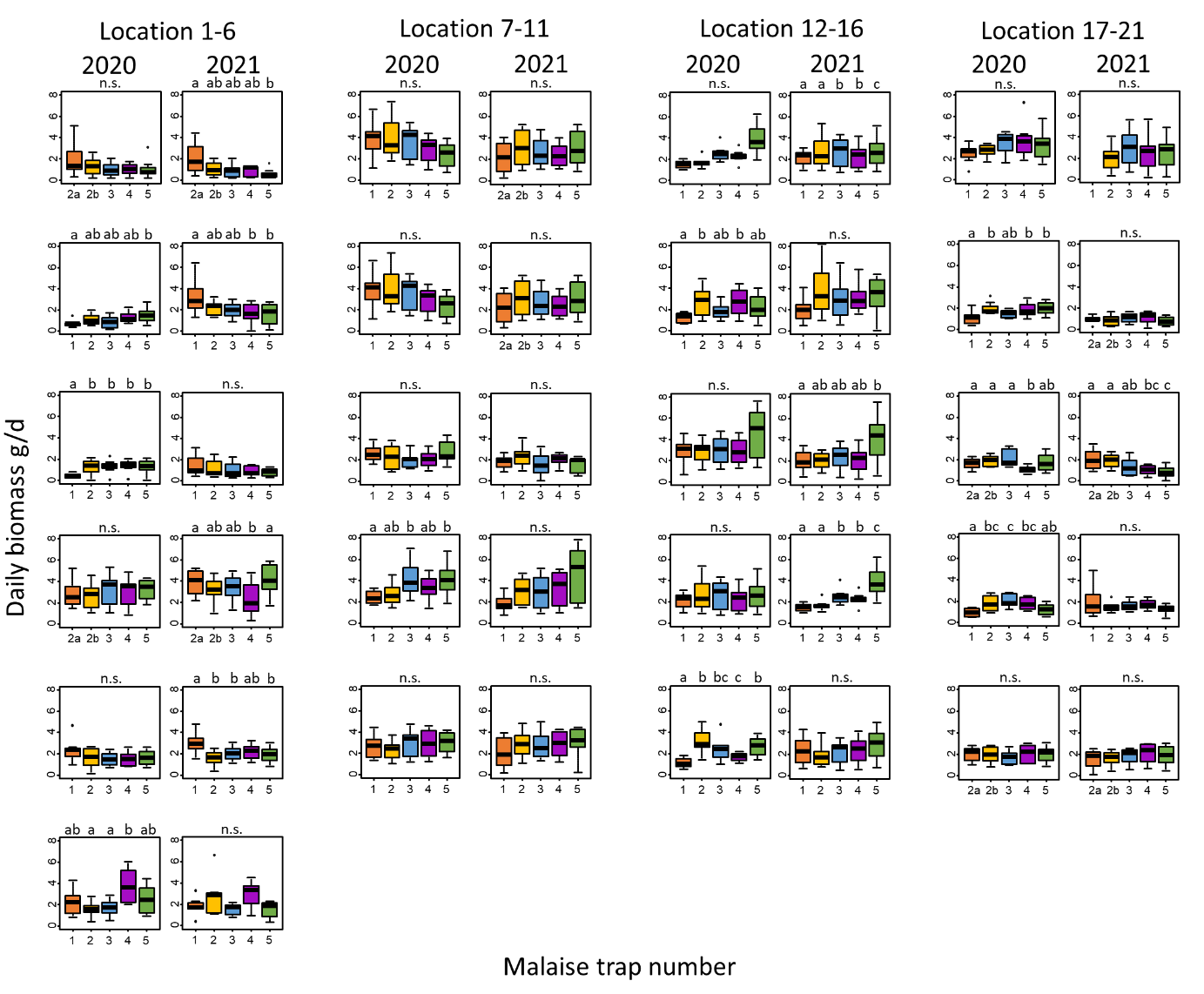
**

**FIGURE S1** Mean daily biomass per trap at each sampling site for 2020 and 2021. Lower case letters classify differing groups with a significance level of p < 0.05 according to post-hoc Mann-Whitney U-tests. Location numbers according to Table S2.


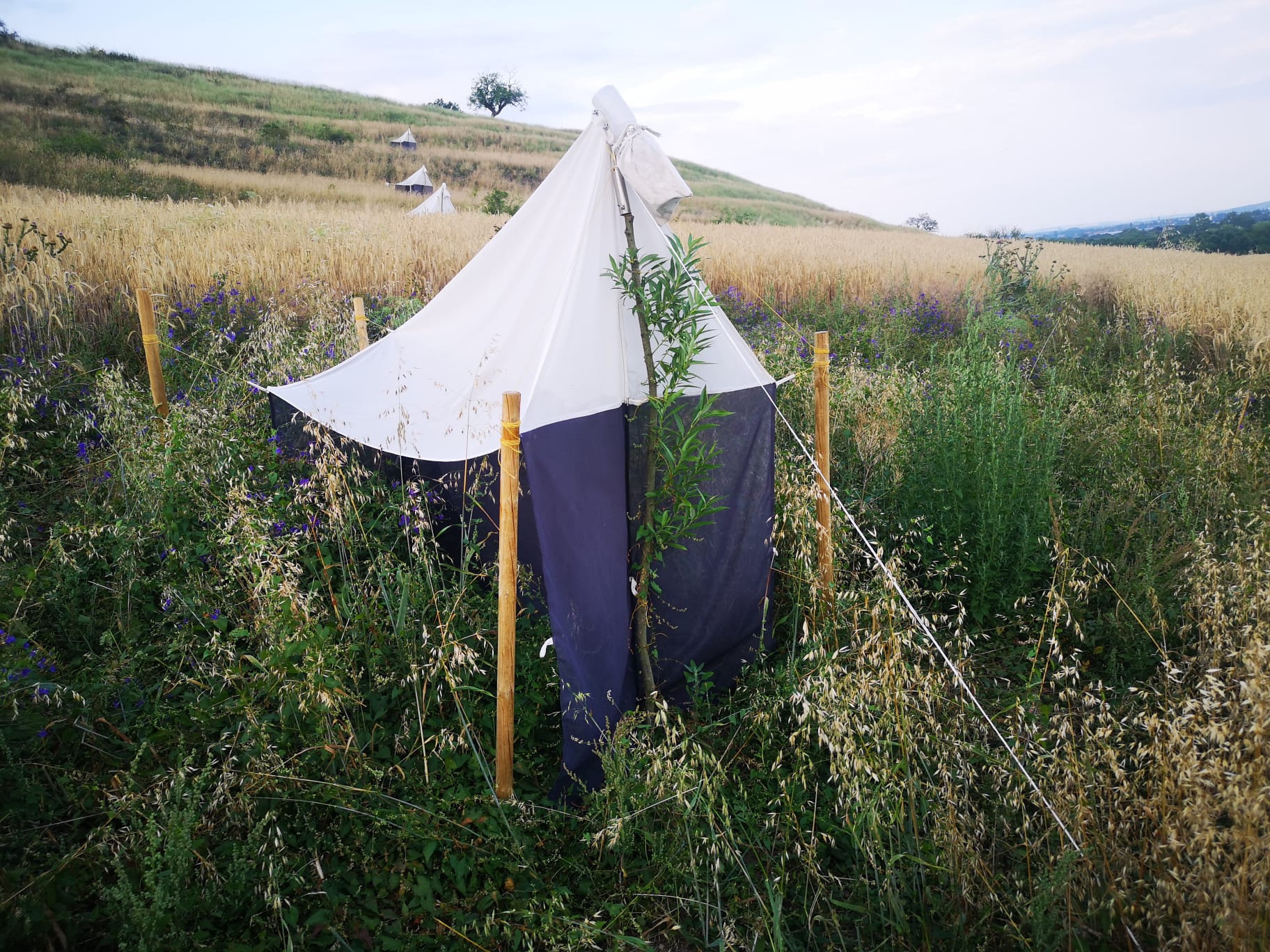


**FIGURE S2** Cereal field with island of wildflowers around Malaise trap 1 (MT1) (Schwellenburg, Thuringia; 26.07.2021)

**TABLE S1** Location information

| **Nr** | **Abbrev.** | **Location** | **State** | **FFH (SAC) -ID *1** | **FFH (SAC) Area [ha] *1** | **NSG-ID *2** | **NSG Area [ha] *2** | **Protected habitat types (the transect is crossing) *3** | **Endangered plant communities along transect (German Red List with status) *4** |
| --- | --- | --- | --- | --- | --- | --- | --- | --- | --- |
| **1** | **LUE** | **Lütjenholmer Heidedünen** | SH | 1320-302 | 313 | 37570 | 18 | Dry sand heaths with Calluna and Genista, Northern Atlantic wet heaths with Erica tetralix | Ericetum tetralicis (2); Myricetum gale (2); Genisto pilosae-Callunetum (2) |
| **2** | **RIE** | **Riedensee** | MV | 1836-301 | 113 | 33182 | 120 | Xeric sand calcareous grasslands |  |
| **3** | **KOO** | **Insel Koos** | MV | 1747-301 | 60406 | 33165 | 1574 | Lowland hay meadows (Corresponding categories: German classification BTT-Nr. 34070102 species-rich, fresh pasture of the planar to submontane level) | Festuco rubrae-Cynosuretum cristati (3); |
| **4** | **GEE** | **Geesower Hügel** | BB | 2752-301 | 82 | 30009 | 39 | Xeric sand calcareous grasslands, Sub-pannonic steppic grasslands | Potentillo arenariae-Stipetum capilaatae (2) |
| **5** | **MAL** | **Oderhänge Mallnow** | BB | 3552-306 | 305 | 30118 | 305 | Xeric sand calcareous grasslands, Sub-pannonic steppic grasslands | Adonido vernalis-Brachypodietum pinnati (2) |
| **6** | **WIS** | **Wisseler Dünen** | NW | 4203-301 | 71 | 35714 | 79 | Inland dunes with open Corynephorus and Agrostis grasslands | Airetum praecocis (3) |
| **7** | **BIS** | **Bislicher Insel** | NW | 4305-301 | 1002 | 36965 | 1053 | Rivers with muddy banks with Bidention-Vegetation, Hydrophilous tall herb fringe communities of plains |  |
| **8** | **GIP** | **Gipskarstlandschaft Hainholz** | NI | 4226-301 | 1327 | 33265 | 641 | Lowland hay meadows | Festuca rubra-Agristis capillaris (3) |
| **9** | **POR** | **Porphyrlandschaft bei Gimritz** | ST | 4437-302 | 819 | 38460 | 288 | Sub-pannonic steppic grasslands, Siliceous rock with pioneer vegetation | Festuco valesiacae-Stipetum capillatae (2) |
| **10** | **ZIE** | **Ziegenbuschhänge bei Oberau** | SN | 4847-301 | 112 | 37981 | 20 | Lowland hay meadows | Arrhenateretum elatioris (V) |
| **11** | **WIP** | **Wipperdurchbruch** | TH | 4631-302 | 6869 | 38140 | 672 | Semi-natural (semi-)dry grasslands and scrubland facies on calcareous substrates (Festuco-Brometalia) | Xerobrometum (3) |
| **12** | **BOT** | **Bottendorfer Hügel** | TH | 4634-303 | 133 | 38143 | 134 | Calaminarian grasslands of the Violetalia calaminariae, Sub-pannonic steppic grasslands | Armerietum bottendorfensis (3) |
| **13** | **SGB** | **Schwellenburg** | TH | 4931-301 | 89 | 38111 | 23 | Rupicolous calcareous or basiphilic grassland of the Alysso-Sedion albi, Sub-pannonic steppic grasslands | Brometum (2) |
| **14** | **HOF** | **Hofberg** | TH | 5327-305 | 260 | 38319 | 43 | Rupicolous calcareous or basiphilic grassland of the Alysso-Sedion albi, Semi-natural dry grasslands and scrubland facies on calcareous substrates (Festuco-Brometalia) | Arrhenateretum elatioris (V); Brometum (2); Gentiano-Koelerietum pyramidatae (3) |
| **15** | **KOP** | **Koppelstein - Helmestal** | RP | 5711-301 | 4551 | 37188 | 87 | Semi-natural (semi-)dry grasslands and scrubland facies on calcareous substrates (Festuco-Brometalia), Lowland hay meadows | Arrhenateretum elatioris (V); Brometum (2) |
| **16** | **DOE** | **Rheinhänge Dorscheider Heide** | RP | 5711-301 | 4551 | 37186 | 626 | European dry heaths, Siliceous rock with pioneer vegetation of the Sedo-Scleranthion | Airo caryophylleae-Festucetum ovinae (3); Thymus-Festuca (3) |
| **17** | **BRA** | **Brauselay** | RP | 5809-301 | 16273 | 37136 | 14 | Stable xerothermophilous formations with Buxus sempervirens on rock slopes (Berberidion), Silicate rocks with pioneer grassland, Tilio-Acerion forests of slopes, screes and ravines |  |
| **18** | **MIT** | **Mittelberg** | BW | 7319-341 | 853 | 30714 | 45 | Semi-natural (semi-)dry grasslands and scrubland facies on calcareous substrates (Festuco-Brometalia) | Brometum (2) |
| **19** | **IPF** | **Ipf** | BW | 7327-341 | 3363 | 30585 | 60 | Juniperus communis formations on calcareous grasslands, Semi-natural dry grasslands and scrubland facies on calcareous substrates (Festuco-Brometalia) | Arrhenateretum elatioris (V); Brometum (2) |
| **20** | **KUE** | **Kürnberg** | BW | 7427-341 | 990 | 30689 | 12 | Juniperus communis formations on calcareous grasslands, Semi-natural (semi-)dry grasslands and scrubland facies on calcareous substrates (Festuco-Brometalia) | Festuca rubra-Agrostis capillaris (3); Brometum (2) |
| **21** | **MUE** | **Mühlhauser Halde** | BW | 7916-311 | 3678 | 31149 | 52 | Juniperus communis formations on calcareous grasslands, Semi-natural (semi-)dry grasslands and scrubland facies on calcareous substrates (Festuco-Brometalia) | Arrhenateretum elatioris (V); Brometum (2) |

*1 https://www.bfn.de/themen/natura-2000/natura-2000-gebiete/steckbriefe.html#c33722

*2 <https://geodienste.bfn.de/schutzgebiete?lang=de&layers=NSG>

*3 EU Commission (2013): EUR 28 Interpretation Manual of European Union habitats

*4 Rennwald, E. et al. (2002). Rote Liste der Pflanzengesellschaften Deutschlands mit Anmerkungen zur Gefährdung. Schriftenreihe für Vegetationskunde. 35. 393-592.


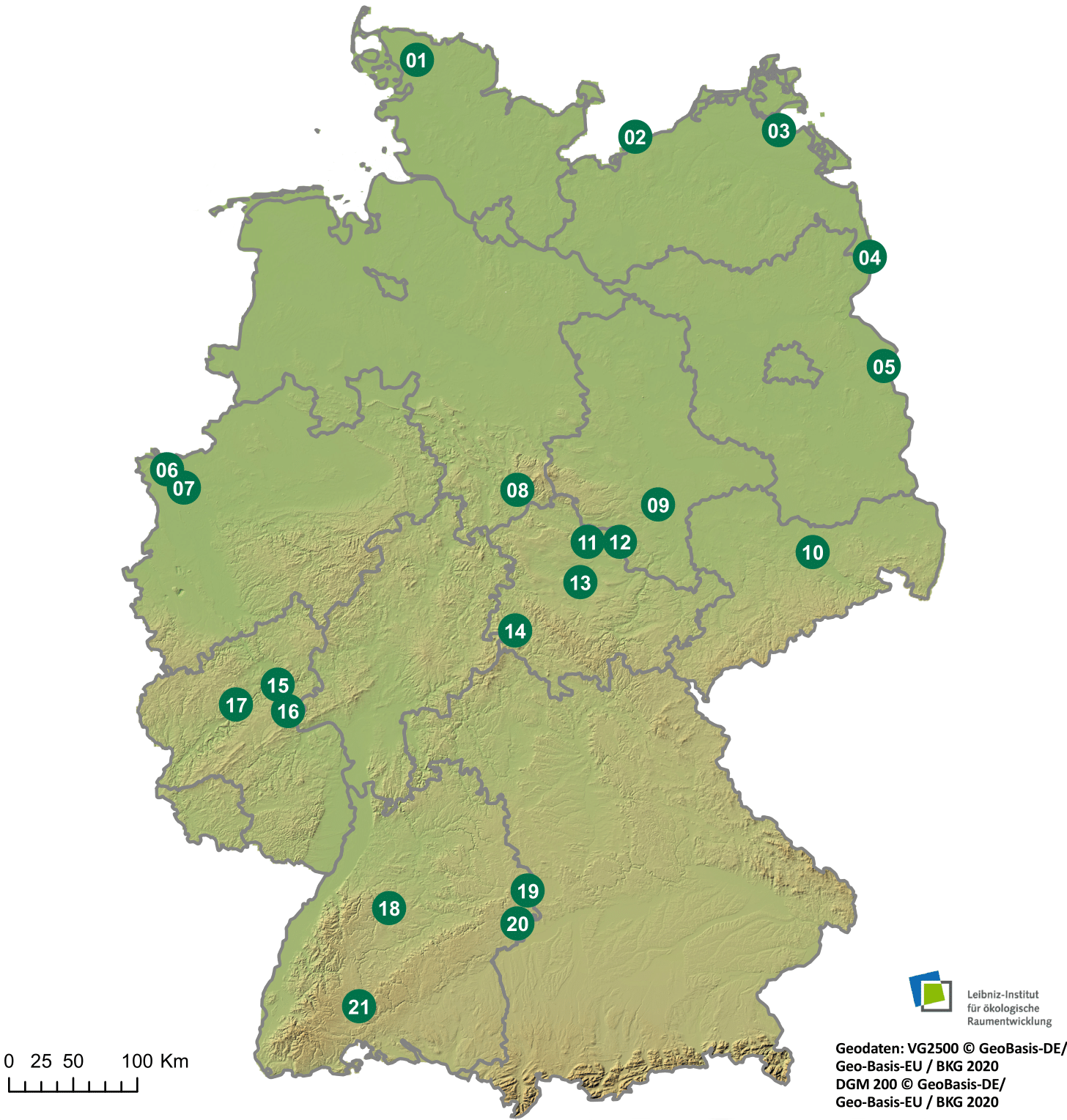


**TABLE S2** Malaise traps sampling interval dates.

| **Interval** | **Dates 2020** | **Dates 2021** |
| --- | --- | --- |
| 1 | 01.05. | 14.05. |
| 2 | 15.05. | 28.05. |
| 3 | 29.05. | 11.06. |
| 4 | 12.06. | 25.06. |
| 5 | 26.06. | 09.07. |
| 6 | 10.07. | 23.07. |
| 7 | 24.07. | 06.08. |
| 8 | 07.08. | 20.08. |

**TABLE S3** R script for applying a GAM analysis.

**>> Install the mgcv package.**

install.packages("mgcv")

**>> Load the mgcv package.**

library(mgcv)

**>> Load your data (or example).**

data<-read.table(file.choose(),header=T)

**>> Assign data for further processing.**

attach(data)

**>> Create GAM model.**

model = gam(daily_biomass_2020 ~ s(temperature_2020, k=3) + s(precipitation_2020, k =3) + s(continental_index, k = 3) + s(arable_land_2km, k=3), data = data , method = "REML", family = gaussian)

**>> Check model for concurvity.**

concurvity(model, full = TRUE)

**>> If concurvity values exceed 0.8 repeat concurvity check of individual variables.**

concurvity(model, full = FALSE)

**>> Check model results.**

summary (model)

**Table S4** Results for the concurvity analysis of the four GAM models.


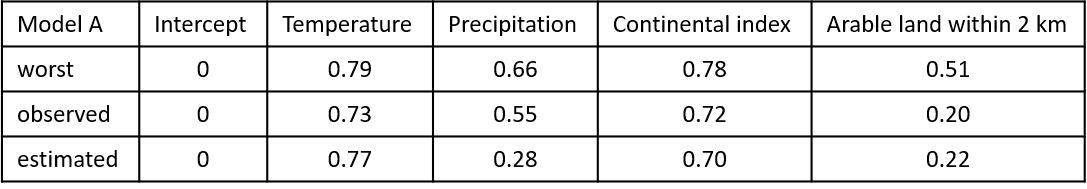


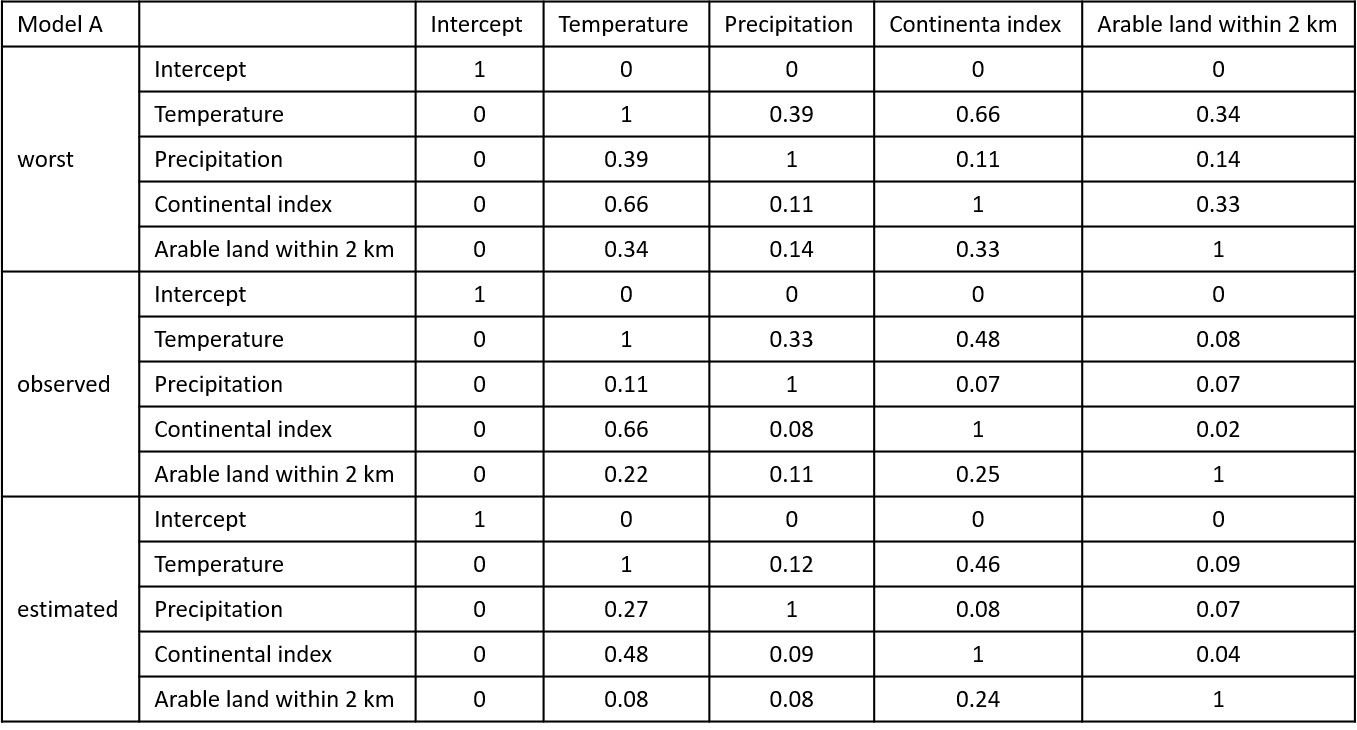


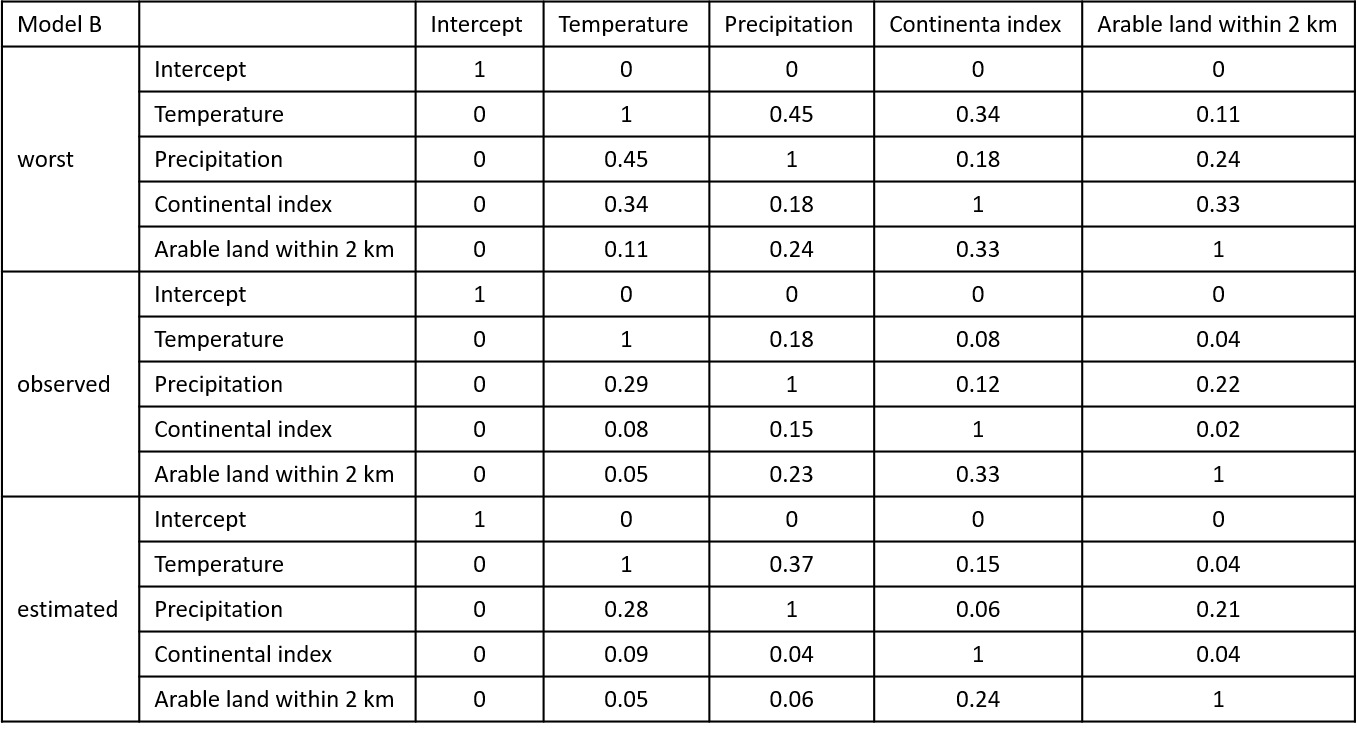


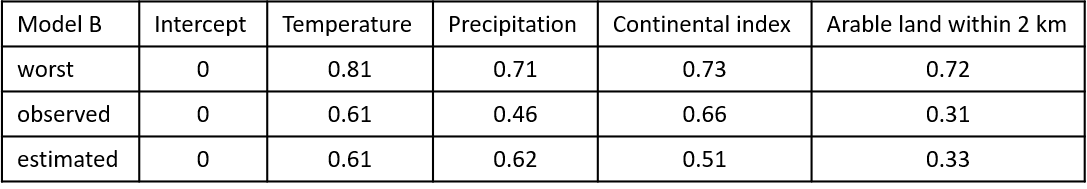


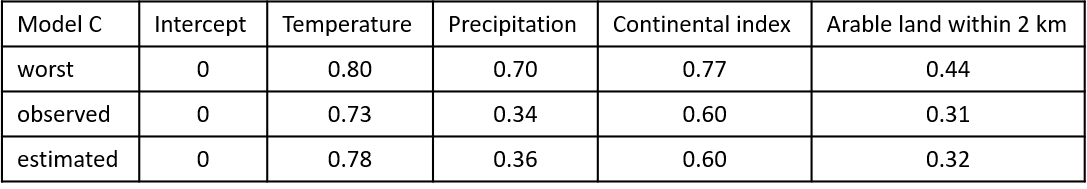


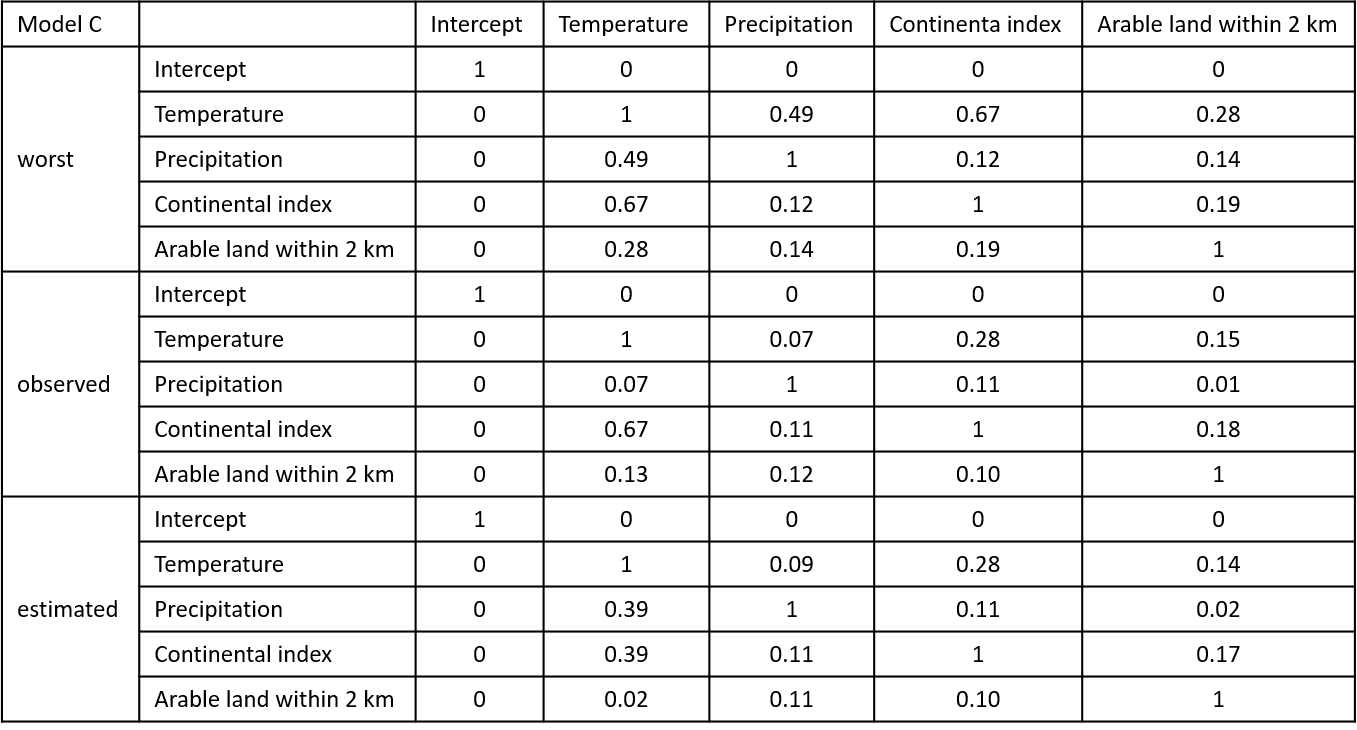


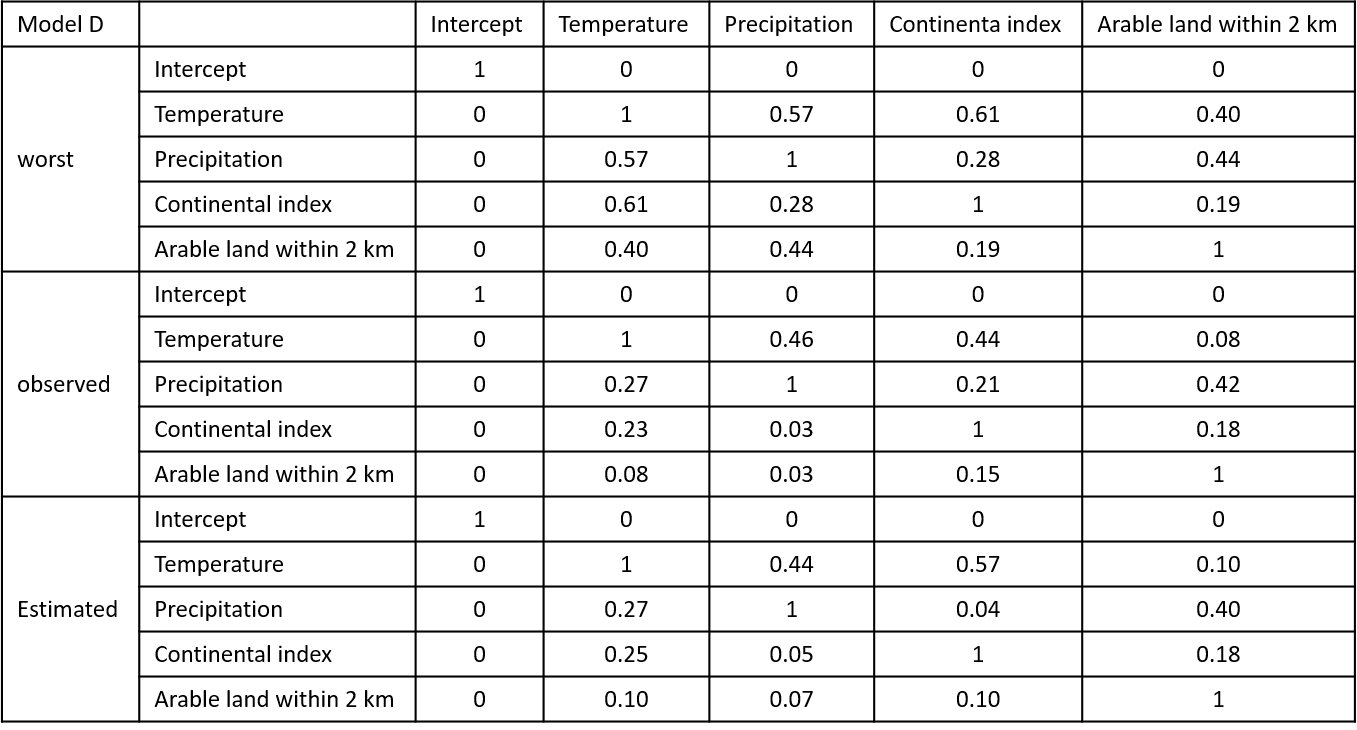

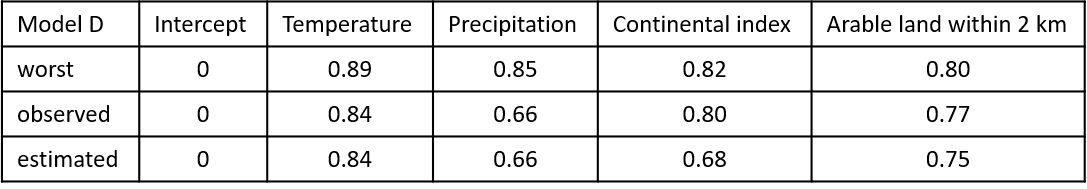


**TABLE S5** Mean daily biomass with standard deviation and sample size (n = total number of insect samples per site) of the two sampling years 2020 and 2021, the percentage difference between the two years.

| **Location** | **Mean daily biomass 2020** | **Mean daily biomass 2021** | **Difference (in %)** | **Site-specific comments** | **Further comments (trap operations)** |
| --- | --- | --- | --- | --- | --- |
| 01_LUE | 1.25 ± 0.99  (n = 40) | 1.14 ± 0.97  (n = 35) | -8.8 | Transect with relocated Malaise trap MT1 in both years | No special observations. Minor damages. |
| 02_RIE | 1.09 ± 0.60  (n = 39) | 2.12 ± 1.14  (n = 40) | +51.4 | Storm damages | No special observations. Team of same three people throughout both seasons. |
| 03_KOO | 1.13 ± 0.66  (n = 39) | 1.10 ± 0.76  (n = 35) | -2.7 | 2021 flower strip at MT1 | Every year with new team. Many damages in spring 2020 due to cattle. Numerous wind damages throughout both years. |
| 04_GEE | 3.00 ± 1.23  (n = 38) | 3.38 ± 1.41  (n = 40) | +11.2 | Transect with relocated Malaise trap MT1 in both years | No specials observations. Minor damages. |
| 05_MAL | 1.72 ± 0.85  (n = 40) | 2.15 ± 0.86  (n= 40) | +20.0 |  | No specials observations. Minor damages. |
| 06_WIS | 2.35 ± 1.36  (n = 40) | 2.10 ± 1.31  (n = 35) | -10.6 |  | Change of trap operator in 2020. Occasionally supported by 07_BIS team. |
| 07_BIS | 3.45 ± 1.43  (n = 40) | 3.62 ± 1.98  (n = 28) | +4.7 | Flooding in 2021 | Team of same people throughout both years. |
| 08_GIP | 3.31 ± 1.58  (n = 39) | 2.67 ± 1.41  (n = 40) | -19.3 | Transect with relocated Malaise trap 1 in 2021 | No special observations. Frequent removal of MT1 due to agricultural activities in 2020. |
| 09_POR | 2.31 ± 0.92  (n = 39) | 1.85 ± 0.86  (n = 36) | -19.9 |  | Numerous storm damages throughout both seasons. Special fixations of traps (bamboo sticks) due to very hard soil. |
| 10_ZIE | 3.34 ± 1.38  (n = 40) | 3.19 ± 1.87  (n = 38) | -4.5 |  | No special observations. Minor damages. |
| 11_WIP | 2.88 ± 1.45  (n = 40) | 2.85 ± 1.66  (n = 39) | -1.0 |  | No special observations. Minor damages. |
| 12_BOT | 1.68 ± 0.98  (n =38) | 1.56 ± 0.68  (n = 38) | -7.1 | 2021 no agricultural practice | Numerous wind damages throughout both seasons. |
| 13_SBG | 2.14 ± 1.18  (n = 40) | 3.06 ± 1.84  (n = 38) | +30.1 |  | Area around MT1 & MT2 not cultivated. Many flowers around traps in 2021. |
| 14_HOF | 3.24 ± 1.61  (n = 39) | 2.50 ± 1.54  (n = 40) | -22.8 |  | Change of trap operator team in spring 2021. |
| 15_KOP | 2.37 ± 1.14  (n = 40) | 2.48 ± 1.22  (n = 40) | +4.4 |  | No special observations. |
| 16_DOE | 2.21 ±1.11  (n = 40) | 2.33 ± 1.22  (n = 40) | +5.1 |  | Change of trap operator team in spring 2021. |
| 17_BRA | 3.16 ± 1.21  (n = 40) | 2.49 ± 1.54  (n = 32) | -21.2 | MT1 missed in 2021 | Very difficult terrain. No special observations. |
| 18_MIT | 1.65 ± 0.65  (n = 40) | 0.95 ± 0.47  (n = 39) | -42.4 | Transect with relocated Malaise trap MT1 in 2021 | Conflict with farmer in summer 2020: relocation of MT1 |
| 19_IPF | 1.73 ± 0.69  (n = 39) | 1.42 ± 0.83  (n = 39) | -17.9 | Transect with relocated Malaise trap MT1 in both years | Change of trap operator in spring 2021. |
| 20_KUE | 1.55 ± 0.70  (n = 40) | 1.65 ± 0.78  (n = 40) | +6.1 |  | No special observations. |
| 21_MUE | 1.95 ± 0.70  (n = 39) | 1.82 ± 0.82  (n = 40) | -6.7 | Transect with relocated Malaise trap MT1 in both years | No special observations. |

**TABLE S6** Distribution of categories I - IV according to the maximum mean biomass. **AL** = arable land, **Bor** = border of arable land and nature reserve, **NPA** = within nature protected area, **Ind** = indistinguishable (at least two categories show similarly high values).

| **Category** | **2020** | **2021** | **Total** |
| --- | --- | --- | --- |
| AL | 1 | 3 | 4 |
| Bor | 6 | 7 | 13 |
| NPA | 8 | 3 | 11 |
| Ind | 6 | 8 | 14 |
|  | 21 | 21 | 42 |

**TABLE S7** Values of the Generalized Additive Models (GAM) from Figure 4.

| Model A | Variable | Edf | F value | P value |
| --- | --- | --- | --- | --- |
|  | Temperature | 1.64 | 1.33 | 0.22 |
|  | Precipitation | 1.47 | 0.54 | 0.64 |
|  | Continentality index | 1.00 | 0.06 | 0.81 |
|  | Agricultural production area in 2 km | 1.00 | 3.81 | 0.07 |
| Model B | Temperature | 1.00 | 0.56 | 0.47 |
|  | Precipitation | 1.36 | 0.27 | 0.76 |
|  | Continentality index | 1.00 | 1.34 | 0.27 |
|  | Agricultural production area in 2 km | 1.00 | 0.13 | 0.72 |
| Model C | Temperature | 1.58 | 0.69 | 0.40 |
|  | Precipitation | 1.00 | 0.35 | 0.57 |
|  | Continentality index | 1.00 | 0.12 | 0.73 |
|  | Agricultural production area in 2 km | 1.00 | 2.65 | 0.13 |
| Model D | Temperature | 1.00 | 0.06 | 0.81 |
|  | Precipitation | 1.00 | 0.52 | 0.49 |
|  | Continentality index | 1.50 | 0.78 | 0.48 |
|  | Agricultural production area in 2 km | 1.00 | 1.08 | 0.32 |

**TABLE S8** Values from the data series in main text Figure 5. DINA data from the years 2020 and 2021 compared to previous data from Hallmann et al. (2017). Significant differences are marked in bold.

| Year | 2020  (n = 829 ) | 2021  (n = 792) |
| --- | --- | --- |
| 1989 (n = 165) | **> 0.001** | **> 0.001** |
| 1990 (n = 63) | **> 0.001** | **> 0.001** |
| 1991 (n = 10) | 0.25 | 0.23 |
| 1992 (n = 56) | **> 0.001** | **> 0.001** |
| 1993 (n = 39) | **> 0.001** | **> 0.001** |
| 1994 (n = 60) | **> 0.001** | **> 0.001** |
| 1995 (n =41) | **> 0.001** | **> 0.001** |
| 1996 (n = 0) | NA | NA |
| 1997 (n = 20) | **0.002** | **0.003** |
| 1998 (n = 0) | NA | NA |
| 1999 (n = 56) | **> 0.001** | **> 0.001** |
| 2000 (n = 47) | **> 0.001** | **> 0.001** |
| 2001 (n = 81) | 0.07 | **0.04** |
| 2002 (n = 0) | NA | NA |
| 2003 (n = 80) | **> 0.001** | **> 0.001** |
| 2004 (n = 48) | **> 0.001** | **> 0.001** |
| 2005 (n = 71) | **0.003** | **0.002** |
| 2006 (n = 26) | **> 0.001** | **> 0.001** |
| 2007 (n = 16) | 0.36 | 0.42 |
| 2008 (n = 24) | 0.90 | 0.84 |
| 2009 (n = 24) | 0.41 | 0.48 |
| 2010 (n = 12) | 0.09 | 0.13 |
| 2011 (n = 4) | **0.02** | **0.02** |
| 2012 (n = 23) | 0.75 | 0.63 |
| 2013 (n = 126) | **0.005** | **0.007** |
| 2014 (n = 348) | **> 0.001** | **> 0.001** |
| 2015 (n = 10) | 0.60 | 0.60 |
| 2016 (n = 62) | 0.35 | 0.30 |
| 2020 | - | 0.86 |
